# Sputtering-deposited amorphous SrVO_x_-based memristor for use in neuromorphic computing

**DOI:** 10.1038/s41598-020-62642-3

**Published:** 2020-04-01

**Authors:** Tae-Ju Lee, Su-Kyung Kim, Tae-Yeon Seong

**Affiliations:** 1 Department of Nanophotonics, Korea University, Seoul, 02841 Korea; 2 Department of Materials Science and Engineering, Korea University, Seoul, 02841 Korea

**Keywords:** Materials science, Materials for devices

## Abstract

The development of brain-inspired neuromorphic computing, including artificial intelligence (AI) and machine learning, is of considerable importance because of the rapid growth in hardware and software capacities, which allows for the efficient handling of big data. Devices for neuromorphic computing must satisfy basic requirements such as multilevel states, high operating speeds, low energy consumption, and sufficient endurance, retention and linearity. In this study, inorganic perovskite-type amorphous strontium vanadate (a-SrVO_x_: a-SVO) synthesized at room temperature is utilized to produce a high-performance memristor that demonstrates nonvolatile multilevel resistive switching and synaptic characteristics. Analysis of the electrical characteristics indicates that the a-SVO memristor illustrates typical bipolar resistive switching behavior. Multilevel resistance states are also observed in the off-to-on and on-to-off transition processes. The retention resistance of the a-SVO memristor is shown to not significantly change for a period of 2 × 10^4^ s. The conduction mechanism operating within the Ag/a-SVO/Pt memristor is ascribed to the formation of Ag-based filaments. Nonlinear neural network simulations are also conducted to evaluate the synaptic behavior. These results demonstrate that a-SVO-based memristors hold great promise for use in high-performance neuromorphic computing devices.

## Introduction

The use of digital computing systems based on von Neumann architecture has led to the development of outstanding memory technologies following Moore’s law over the last several decades, with a consistent aim to manufacture integrated circuits with a higher number of transistors and a lower energy consumption within a smaller area. To achieve this, high-density and low-power computing system engineering is essential, but traditional Si-based devices face a number of physical limitations^1^, thus innovative computing architecture is required. In this vein, neuromorphic computing systems inspired by the human brain have been introduced and are considered a promising approach to overcoming the bottlenecks associated with conventional von Neumann computing systems^2–4^.

To construct neuromorphic computing systems, artificial memristor-based synapses that have their synaptic characteristics updated by electrical stimuli are arranged in a circuit^5,6^. With this structure in mind, resistive random access memory (RRAM) has emerged as a highly promising contender for use in future computing devices due to its great scalability, low energy consumption, quick switching (sub-ns), and simple two-terminal structure with a cell size of 4*F*^2^, where *F* is the minimum feature size^1,7–10^. Extensive research has thus been conducted on RRAM to take advantage of its high storage density and potential application in neuromorphic computing systems. Most resistive switching materials in RRAM exhibit two states: a low resistance state (LRS) and a high resistance state (HRS). This type of multilevel resistance allows the possibility of multilevel storage, which can provide more storage space within a single cell and generate synaptic behavior, leading to material and structural innovation^11–14^. Thus, researchers have made an effort to realize multilevel storage using a variety of approaches, including heterostructures^15,16^, the insertion of an interlayer^17,18^, and doping techniques^19,20^.

Various materials have been proposed for the active layer, such as binary oxides^21–23^, perovskite materials^24–28^, and organic materials^29,30^, and these materials exhibit different types of resistive switching mechanism under certain conditions. For instance, Pt/TiO_2_/Pt devices have been found to exhibit unipolar resistive switching behavior^31^, but modulation of the polarity of the electroforming in Pt/TiO_2_/Pt leads to bipolar reset^32^. In particular, perovskite-based switching behavior has been widely investigated because of the distinctive properties of this material. Most previous studies of perovskite-based RRAM have focused on metal-to-insulator (MIT) materials^33,34^. For example, bulk ABO_3_ has been reported to have a metallic nature with low electrical resistivity^35^, with the Hubbard model indicating that MIT systems behave as a Mott insulator under critical conditions^35–37^. MIT materials such as Ca_2_RuO_4_, (V_1−*x*_Cr_*x*_)_2_O_3_, and Pr_0.7_Ca_0.3_MnO_3_ have been regularly investigated and have proven to be capable of resistive switching^38–40^. However, switching behavior can only be realized with high-temperature treatment and the use of specific substrates during fabrication, meaning these materials are unsuitable for complementary metal-oxide semiconductor (CMOS) processes, hampering the development of commercial MIT-based devices. For practical applications, fabrication has to be carried out at low temperatures, and CMOS-compatible substrates need to be employed. In this respect, it has been shown that nonstoichiometric perovskite-type oxides synthesized at room temperature can exhibit defect-related resistive switching characteristics, a general property of RRAM devices^25,41^.

In this study, we characterized the electrical performance of a metal-insulator-metal (MIM) structure in which an a-SVO thin film was sandwiched between bottom Pt and top Ag electrodes and evaluated its resistive switching characteristics at room temperature. Note that a perovskite-structured SrVO_3_ single-crystal thin film was used as a transparent conducting electrode because of its high electrical conductivity and high transmittance^42^. As compared to crystalline SrVO_3_, the sputtered a-SVO film has advantages, including large area thickness and composition uniformity, low temperature growth, structural and compositional variations by engineering the oxygen content^43^, excellent optical transparency in the near-ultraviolet (UV) region^44^, and outstanding flexibility. The a-SVO films exhibit a large bandgap and low electrical conductivity because V *3d* orbitals dominantly affect the in-gap states near the Fermi energy, and the substantial distortion in the V-O6 octahedra causes an increase in the separation between the *d*_||_ orbitals and the π^*^ band^43–45^. Our sputtered a-SVO film, which acted as an insulator, displayed nonvolatile resistive switching behavior, and reversible multilevel transitions were able to be controlled using voltage modulation. In addition, the synaptic behavior of the a-SVO film was investigated as a function of voltage stress in order to determine the suitability of the proposed a-SVO-based memristor for use in neuromorphic computing.

## Results and Discussion

### Nonlinear cyclic voltammetry of the a-SVO memristor

**Figure 1:**
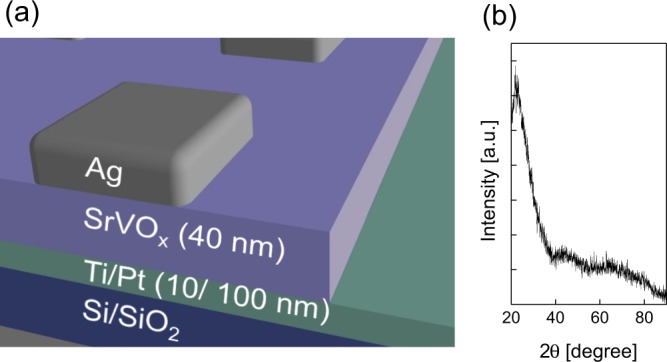
(**a**) 3D schematic diagram of an as-fabricated Ag/a-SVO/Pt memristor. (**b**) Typical *θ*-2*θ* XRD pattern of a 100-nm-thick a-SVO thin film deposited on a glass substrate at room temperature.

Fig. 1(a) displays a schematic diagram of the proposed a-SVO memristor. A 40-nm-thick a-SVO active layer is sandwiched between a Pt film as the bottom grounding electrode and an Ag film as the top terminal electrode (see the Experimental Section for more details). To examine the microstructure of the sputtered a-SVO, X-ray diffraction (XRD) analysis of a 100-nm-thick a-SVO thin film on a glass substrate was conducted (Fig. 1(b)). The XRD results revealed a broad intense peak at *θ* = ~22°, which is characteristic of the amorphous phase. Furthermore, Hall measurements showed that the a-SVO film had extremely high resistance at room temperature, indicating the lack of mobile charge carriers.

**Figure 2:**
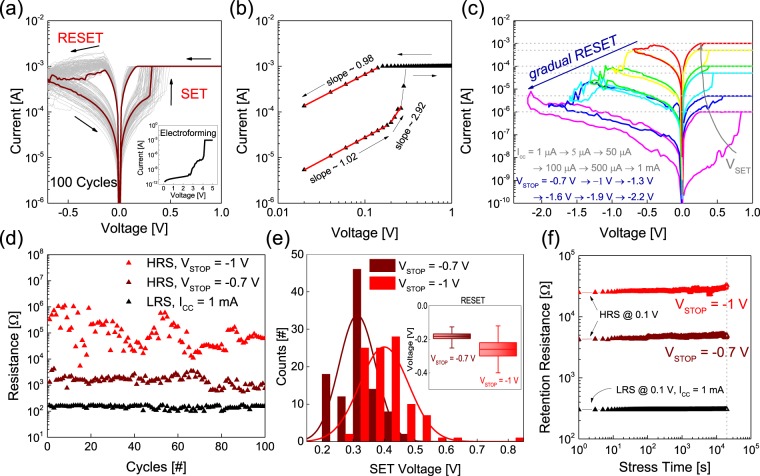
An a-SVO-based memristor exhibiting bipolar resistive switching. (**a**) *I-V* properties of the Ag/a-SVO/Pt memristor after 100 consecutive DC cycles. The inset graph represents the electroforming process. (**b**) DC *I-V* properties with a positive bias and the corresponding slopes on a double-logarithmic scale. (**c**) *I*_CC_-modulated DC *I-V* curves with a positive bias and stop voltage-modulated DC *I-V* curves with a negative bias. The arrow denotes the sweep sequence. **(d**) Endurance testing up to 100 cycles at an LRS with an *I*_CC_ of 1 mA and at HRSs with *V*_STOP_ = −0.7 and −1 V. (**e**) The distribution of the SET voltage. The inset indicates the distribution of the RESET voltage. (**f**) Retention testing up to 2 × 10^4^ s at an LRS with an *I*_CC_ of 1 mA, and at HRSs with *V*_STOP_ = −0.7 and −1 V.

Fig. 2(a) presents the *I-V* curves of an Ag/a-SVO/Pt device derived from measurements taken at room temperature using a DC double ramp sweep for 100 cycles. To operate the a-SVO memristor, electroforming was conducted using voltage sweeping from 0 to 5 V with a compliance current (*I*_CC_) of 1 mA, as shown in the inset of Fig. 2(a). The *I-V* characteristics during the voltage sweeps are indicative of typical bipolar resistive switching behavior^15–20^. After the electroforming process, DC voltage sweeping from 0 V → 1 V → 0 V with an *I*_CC_ of 1 mA resulted in a SET process (i.e., an off-to-on transition) and sweeping from 0 V → −0.7 V → 0 V without an *I*_CC_ led to a RESET process (i.e., an on-to-off transition). A rapid increase in the current from an HRS to an LRS was observed for the SET process, while a gradual decrease in the current occurred from an LRS to an HRS for the RESET process without an *I*_CC_. It is important to note that, in the RESET process, the a-SVO device needed a higher negative current than the required *I*_CC_, which has been observed in previous results^46^. It is noted that variation of the resistances obtained from four randomly selected devices after 10 cycles (Fig. S1) indicates the uniform performance of different Ag/a-SVO/Pt-based devices.

To understand the conduction mechanism of the a-SVO memristor, a double-logarithmic scaled *I-V* curve was obtained (Fig. 2(b)). The fitted curve of the a-SVO memristor exhibited conduction behavior that was consistent with a typical trap-assisted space-charge-limited current^47^. In particular, three distinct regions representing different conduction mechanisms are observable in Fig. 2(b). In the low voltage region (<0.2 V), the slope of the fitted line is approximately 1.02 $(I\propto {V}^{1.02})$, which indicates Ohmic conduction. The region between 0.2 and 0.27 V has a slope of about 2.92 $(I\propto {V}^{2.92})$, indicating that Child’s law conduction is dominant. The third region between 0.27 and 0.3 V has a slope of over 14. These conduction mechanisms are typical of the phenomena observed for bipolar resistive switching devices^48^.

In order to emulate a synaptic device, the multilevel resistance properties of the a-SVO memristor were examined as a function of *I*_CC_ in the range of 1 μA–1 mA and sweep stop voltages (*V*_STOP_) in the range of −0.7 V to −2.2 V (Fig. 2(c)). Multilevel resistance states, i.e., analog switching behavior, were observed in the SET and RESET processes, which are essential electrical properties for neuromorphic computing applications^49^. Fig. 2(d) displays the resistance (corresponding to either LRS or two HRSs) as a function of the number of cycles. The LRS at *I*_CC_ = 1 mA and the HRS at *V*_STOP_ = −0.7 V exhibit uniform resistance with little deviation. On the other hand, the resistance of the HRS at *V*_STOP_ = −1 V fluctuated significantly with an increasing number of cycles up to about 90, after which the variation becomes lower.

Fig. 2(e) presents the classical Gaussian distribution of the SET voltages for the two HRSs of the a-SVO memristor. The cycle-to-cycle variation of the HRS at *V*_STOP_ = −1 V is larger than that of the HRS at *V*_STOP_ = −0.7 V. This could be related to the fact that the former is more strongly affected by the thermal effect caused by the higher electric field. The variation in the RESET voltage (*V*_RESET_) is proportional to the distribution of the SET voltage (*V*_SET_), as shown in the inset of Fig. 2(e). Fig. 2(f) presents the retention test results of the a-SVO memristor for different resistance states measured at room temperature. Individual states were monitored at a read voltage of +0.1 V every 2 s, and no significant changes were observed for a period of 2 × 10^4^ s. This is because the atomic migration of a conductive filament due to Joule heating rarely occurs under voltage stress^50^. These results indicate that the a-SVO memristor satisfies the requirements for use in potential synapse devices.

### Conduction mechanisms for the a-SVO memristor

**Figure 3:**
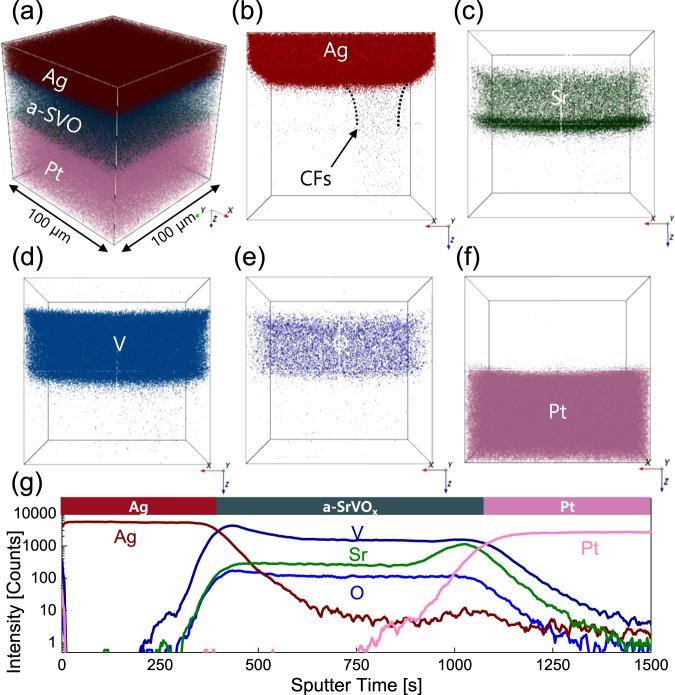
ToF-SIMS depth profiles of the Ag/a-SVO/Pt memristor after cyclic voltammetry. (**a**) Merged 3D mapping image of Ag, Sr, V, O, and Pt signals. (**b–f**) 3D mapping images of Ag, Sr, V, O, and Pt. A cone-shaped Ag conduction filament is observed in the a-SVO film, as marked by the dotted lines. (**g**) Depth profiles showing the distribution of the selected elements.

Time-of-flight secondary ion mass spectroscopy (ToF-SIMS) 3D mapping was utilized to determine the mechanisms responsible for the formation of the conductive filament because it represents a powerful tool for visualizing target behavior within complex structures^51^. Three-dimensional mapping of th Ag/a-SVO/Pt memristor was achieved via rasterizing over a 100 × 100 μm^2^ area using a Cs^+^ beam with an energy of 3 keV and a current of 30 nA after DC sweeping at *I*_CC_ = 1 mA and *V*_STOP_ = −0.7 V for 100 cycles. Fig. 3(a)–(f) illustrates the spatially resolved 3D mapping images of the Ag/a-SVO/Pt memristor, with the color-coded element signals corresponding to Ag, Sr, V, O, and Pt. Fig. 3(g) displays the depth profiles of the Ag/a-SVO/Pt memristor, which was stressed using an electric field at room temperature. The mapping results revealed that Ag atoms had drifted through the a-SVO thin film and connected the top Ag electrode to the bottom Pt one (Fig. 3(b)). The conductive filament is depicted as a cone-shaped rod, with the diameter of the filament larger as it gets closer to the Ag electrode.

**Figure 4:**
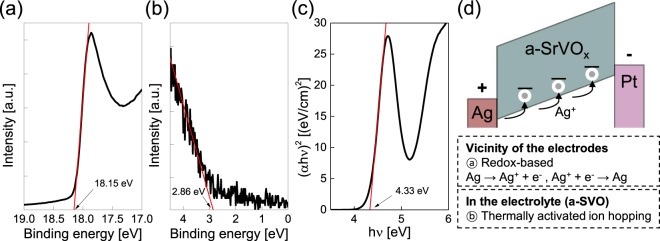
UPS analysis of an a-SVO film. (**a**) UPS SECO region and (**b**) valence band edge region of the a-SVO film. (**c**) Tauc plot from UV-Vis analysis of the a-SVO film. The red line represents the fitting line in the linear region. (**d**) Schematic energy band diagram of the Ag/a-SVO/Pt memristor under forward voltage bias. The conduction of Ag atoms is dominated by hopping from traps near the valence band edges of the a-SVO film, which is pinned by the electrodes. At the electrode/a-SVO interfaces, redox-based reactions occur, where Ag atoms are ionized into Ag^+^ ions or the Ag^+^ ions are reduced back to Ag atoms.

Fig. 4(a,b) presents the ultraviolet photoelectron spectroscopy (UPS) secondary electron cut-off (SECO) region and the valence band edge region, respectively of the a-SVO film on an Si substrate. The work function of the a-SVO thin film can be directly estimated using the SECO point by line-fitting the low kinetic energy and valence band edge regions^52^. To produce a band diagram for the Ag/a-SVO/Pt structure, the optical band gap energy was determined using the Tauc relation (*αhν*)^2^ vs *hν*, where *α* is the absorbance coefficient gained from UV-visible measurements, which is an accepted method for evaluating the optical band gap energy of amorphous materials^53^ (Fig. 4(c)). The optical bandgap and work function were calculated to be 4.3 eV and 3.1 eV, respectively. The electron affinity of the a-SVO film was also evaluated to be 1.6 eV using UPS. Our findings were consistent with previous results^44^, with the optical transmittance of crystalline SVO films rapidly dropping at wavelengths below 520 nm; however, the sputtered a-SVO thin films grown at room temperature did not experience a reduction in transmittance even at wavelengths below 400 nm.

Based on the band gaps, work functions, and electron affinities, Fig. 4(d) presents a band diagram of the Ag/a-SVO/Pt memristor. High double-Schottky barriers formed at the interfaces between the bottom Pt electrode (work function = 5.3 eV), the a-SVO film, and the top Ag electrode (4.7 eV). When a positive voltage was applied to the top Ag electrode and the bottom Pt one was grounded, the electric field was driven by the difference in the potential. With increasing voltage, Ag ions gradually appeared and dissolved into the a-SVO film, acting as an electrolyte. This amorphous electrolyte contained numerous point defects, trapping Ag^+^ or e^−^. The electric field caused these Ag^+^ ions to drift toward the bottom Pt electrode in an ion-hopping process^4^. The deposited Ag atoms then grew from the bottom Pt electrode to the Ag top electrode, forming a filament^2,6,9^. Consequently, the filament connected the two electrodes, converting the resistance of the a-SVO memristor from an HRS to an LRS (i.e., instigating the SET process). The thickness of the conductive filament can be controlled by modulating the current through the memristor, which means the conductance is adjustable. This feature is an essential requirement for a memristor to serve as a synaptic device.

**Figure 5:**
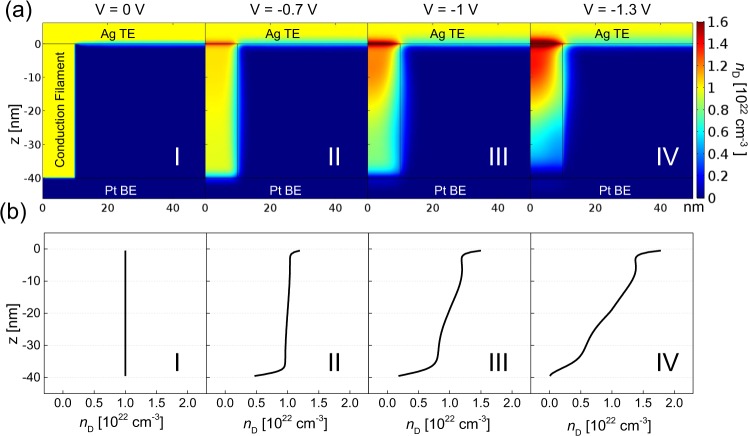
COMSOL Multiphysics simulation results for the RESET process. (**a**) Calculated 2D images of *n*_D_ and (**b**) 1D profiles for states I (0 V), II (−0.7 V), III (−1 V), and IV (−1.3 V).

A COMSOL Multiphysics s imulation was also carried out to further investigate the conduction mechanism involved in the Ag/a-SVO/Pt memristor^54–56^. To understand the RESET process, the thermal and electrical effects were taken into account in the simulations. The RESET process can be described by the following equations:

1 $$\frac{{\rm{\partial }}{n}_{{\rm{D}}}}{{\rm{\partial }}t}={\rm{\nabla }}\cdot (D{\rm{\nabla }}{n}_{{\rm{D}}}-\mu {n}_{{\rm{D}}}{\rm{\nabla }}\psi )$$

2 $$\nabla \cdot \sigma \nabla \psi =0$$

3 $$-\nabla \cdot {k}_{{\rm{th}}}\nabla T=\sigma {|\nabla \psi (r,z)|}^{2},$$

where *n*_D_, *D*, *μ*, *ψ*, *σ*, and *k*_th_ are the concentration of Ag atoms, the diffusivity, the drift velocity, the external applied voltage at the Ag electrode, the electrical conductivity, and the thermal conductivity, respectively. In the simulation, the conductive filament and top Ag electrode were assumed to have a uniform Ag concentration (*n*_D_) of 1 × 10^22^ cm^−3^ along the direction of the electric field direction (i.e., the filament) for the SET state. When the DC voltage was increased, the current flowed locally through the conductive filament and the device temperature increased because of Joule heating. The bias-induced thermal energy lowered the potential energy for the hopping of Ag^+^ ions, enhancing Ag migration along the electric field. Fig. 5(a) presents 2D maps for *n*_D_ at reverse biases ranging from 0 to −1.3 V, while Fig. 5(b) displays 1D profiles for *n*_D_ along the z-axis (i.e., the conductive filament) during the gradual onset of the RESET process. As can be seen in the simulation results, the concentration of Ag atoms in the vicinity of the bottom Pt electrode decreased with increasing reverse bias, reducing the electrical conductivity of the a-SVO memristor. Consequently, the resistance of the memristor increased further during the RESET process.

### Simulating the synaptic behavior of the a-SVO memristor

**Figure 6:**
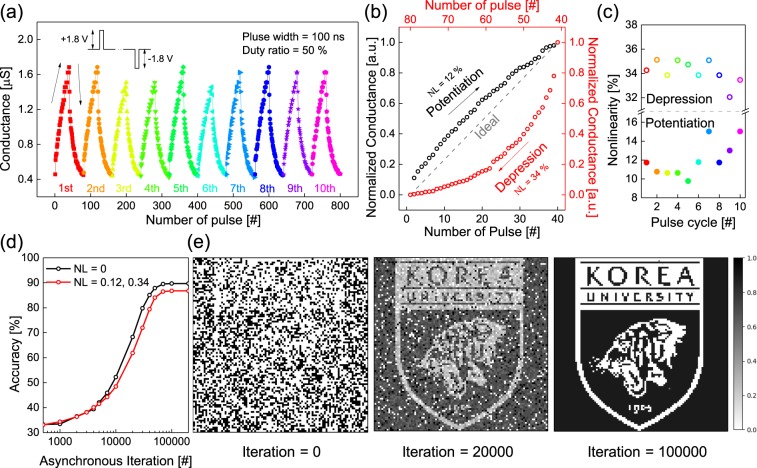
(**a**) 10 cycles and (**b**) normalized conductance of potentiation/depression synaptic plasticity of the Ag/a-SVO/Pt memristor. The nonlinear fitting curves can be described using the equation $$G(n)={G}_{initial}+{A}_{1}$$$$[1-\exp (-n/{\tau }_{1})]+{A}_{2}[1-\exp (-n/{\tau }_{2})]$$. (**c**) NL values of the potentiation and depression processes. (**d**) The simulated accuracy and (**e**) synaptic weight maps of a 100 × 100 binary image as a function of asynchronous iterations. Two schemes are calculated: the ideal case (NL = 0) and our device (NL = 0.12 and 0.34).

To mimic the synaptic behavior of the a-SVO memristor, its conductance transition characteristics were examined by modulating the bias (Fig. S2). Ten cycles consisting of 40 repeated potentiation pulses (*P*-pulses) at 1.8 V with a pulse width of 100 ns followed by 40 repeated depression pulses (*D*-pulses) at −1.8 V with a pulse width of 100 ns were applied to the a-SVO memristor. The readout of the current was simultaneously carried out for each pulse, with the duty ratio of all pulses fixed at 50%. The analog switching behavior, which exhibits a gradual conduction transition with minimal fluctuation, is shown in Fig. 6(a). The conductance of the a-SVO device increases with the *P*-pulses and decreases with the *D*-pulses. It can be seen that 40 weight states can be accurately controlled according to the number of *P*- or *D*-pulses. The conductance can be tuned by varying the amplitude, the width, the duty ratio, and the number of pulses. Moreover, for neuromorphic computing applications, lowering the energy consumption per synaptic spike (*V*^2^ × *t* times the conductance) of the memristor is a crucial requirement^57^. Thus, to develop synaptic devices with fast programming speeds, the pulse conditions need to be optimized. Fig. 6(b) presents the normalized conductance as a function of the number of *P*- or *D*-pulses. At both voltages, nonlinear normalized conductance behavior can be observed, with the voltage with a higher negative bias exhibiting a larger variation. Furthermore, the nonlinearity (NL) values for the depression and potentiation processes were characterized (Fig. S3). The NL values for the depression process (0.32–0.35) were larger than those for the potentiation process (0.10–0.15), as shown in Fig. 6(c). This indicates that the rupture of the conductive filament takes place more rapidly than its formation, which involves a more complicated process^58^.

To evaluate the associative memory of the a-SVO memristor for neuromorphic system applications, a Hopfield neural network (HNN) simulation^59^, which has been commonly used to investigate associative memories and parallel processing, was conducted using Python. With associative memory, certain received patterns can be stored in the form of connection strength within the neural network. Therefore, stored patterns can be successfully retrieved through associated states. All synapses of an HNN are fully connected so that each synapse has a direct connection with all other synapses. These connections have an associated connection strength between the synapses. In the HNN simulation in the present study, a sigmoid activation function was used to metamorphose the graded weight updates of the synapses for nonlinear synaptic behavior^60^. In the calculation, 100 × 100 synapses were used (i.e., a total of 10,000), which were trained by a simple binary image (black = 1 and white = 0) whose initial conductance was the noised normalized conductance. The input patterns were assumed to be polarized, and the weight matrix for the input pattern was calculated using Eq. (4) below:

4 $$W=\mathop{\sum }\limits_{i=1}^{n}{X}_{i}{X}_{i}^{T}-nI,$$

where *X*_*i*_ is the vector constructed from the *i*^th^ input pattern, *W* is the weight matrix of the input patterns, *n* is the number of patterns, and *I* is the unit matrix. The input patterns were also converted into a vector and multiplied. The trained synapses were asynchronously potentiated or depressed by weight updates receiving binary information. This operation was randomly selected, representing asynchronous updating, in order to mimic biological synapses. An ideal case in which the value of NL was 0 was also calculated to evaluate the characteristics of the a-SVO memristor. The accuracy of the calculations compared to the ideal case was determined using Eq. (5):

5 $$Accuracy\, \% =\sqrt{{\sum }_{i=1}^{m}\frac{{({G}_{target}(i)-{G}_{trained}(i))}^{2}}{m}}\times 100\, \% ,$$

where *G*_*target*_(*i*) and *G*_*trained*_(*i*) are the target and trained normalized conductance of the *i*^*th*^ synapses, respectively, and *m* is the total number of synapses (Fig. 6(d)). In the ideal case, the accuracy was saturated at 89.7% within 100,000 iterations, while the accuracy of our simulated device was saturated at 86.8%. The reason why the accuracy of the ideal case was not saturated at 100% could be that, in our simulation, a sigmoid function was adopted, which produces a non-binary response, or a Hopfield network energy that is localized at certain minimum energy states^61,62^. The synaptic weight maps of 100 × 100 synapses for 0, 20,000, and 100,000 asynchronous iterations when NL = 0.12 for potentiation and NL = 0.34 for depression are presented in Fig. 6(e). These results show that the proposed a-SVO memristor can be successfully trained to recognize the input pattern.

## Conclusion

In summary, a sputtered a-SVO film sandwiched between top Ag and bottom Pt electrodes successfully demonstrated nonvolatile multilevel resistive switching and synaptic behavior under DC and pulse voltage stresses. In particular, analog switching, an essential characteristic for use in synaptic devices, was realized by modulating the *I*_CC_ in the forward bias and the *V*_STOP_ in the reverse bias. Based on the results of ToF-SIMS, UPS, and the COMSOL Multiphysics simulation, this conduction was attributable to the formation of Ag-based filaments. Nonlinear HNN simulations were also conducted to evaluate the synaptic properties of the a-SVO memristor. These results indicate that room-temperature sputtered a-SVO films have the potential for use in high-performance nonvolatile memory or neuromorphic computing devices, and offer the possibility of using amorphous inorganic perovskite for information storage.

## Methods

### a-SVO memristor fabrication process

To fabricate the proposed a-SVO-based RRAM device, a commercial wet-oxidized SiO_2_ (300 nm)/Si substrate was adopted. This substrate was cleaned with acetone, methanol, and deionized water to remove contaminants from the surface for 5 min per cleaning agent. A 100-nm-thick Pt thin film was then deposited as the bottom electrode, followed by a 10-nm Ti adhesion layer, using e-beam evaporation. This was followed by a 40-nm-thick a-SVO thin film, which was deposited using an RF sputtering system with a 2-inch polycrystalline SrVO_3_ target (atomic ratio Sr:V:O = 1:1:3). The a-SVO thin film was deposited at room temperature under the following conditions: an RF sputtering power of 60 W, a working pressure of 1.33 Pa, and an Ar flow rate of 23 sccm. Finally, a 150-nm-thick top Ag electrode was added using RF sputtering with a patterned shadow mask.

### Electrical measurements

A standard DC voltage sweep and retention test for multilevel states in the proposed Ag/a-SVO/Pt memristor was conducted using an Agilent 4155 C Semiconductor Parameter Analyzer, with the voltage bias applied to the top Ag and bottom Pt electrodes at room temperature. Pulse measurement was performed using a pulse generator (Agilent 81101 A) with a four-channel oscilloscope (Hantek 6254bd).

### Optical and chemical characterizations

To plot the Tauc relation, the absorbance of a 100-nm-thick a-SVO film on a sapphire substrate was measured using a UV-visible spectrometer (UV-1800, Shimadzu) in the range of 200–900 nm. The crystal structure of the a-SVO was assessed with X-ray diffraction (XRD, SmartLab, Rigaku) in the range of 20–90° at a speed of 5°/min with a step size of 0.02°. A Cu target (λ = 1.5412 Å) was used as the X-ray source. The work function of the a-SVO was determined using UPS (AXIS SUPRA, Kratos). To investigate the conductive filament, ToF-SIMS (TOF.SIMS-5, ION-TOF, Münster, Germany) was used with a rastered Cs^+^ beam with an energy of 3 keV and a current of 30 nA (raster size: 300 μm × 300 μm).

## Supplementary information

supplementary information.
