# Supplementary material for: Sputtering-deposited amorphous SrVOx-based memristor for use in neuromorphic computing
Source: Sci Rep. 2020 Apr 1;10:5761. doi: 10.1038/s41598-020-62642-3 (PMC7113278; doi:10.1038/s41598-020-62642-3)
Supplement: Supplementary file 1 — supplementary information. [file 41598_2020_62642_MOESM1_ESM.pdf]

Supplementary data for

Sputtering-deposited amorphous $SrVO_x$-based memristor for use in neuromorphic computing

Tae-Ju Lee[1], Su-Kyung Kim[2], and Tae-Yeon Seong[1,2,*]

[1]Department of Nanophotonics, Korea University, Seoul 02841, Korea

[2]Department of Materials Science and Engineering, Korea University, Seoul 02841, Korea

*tyseong@korea.ac.kr

1. Device variation

The resistance distribution of four randomly selected devices (10 cycles for each device), supplementary Fig. S1.

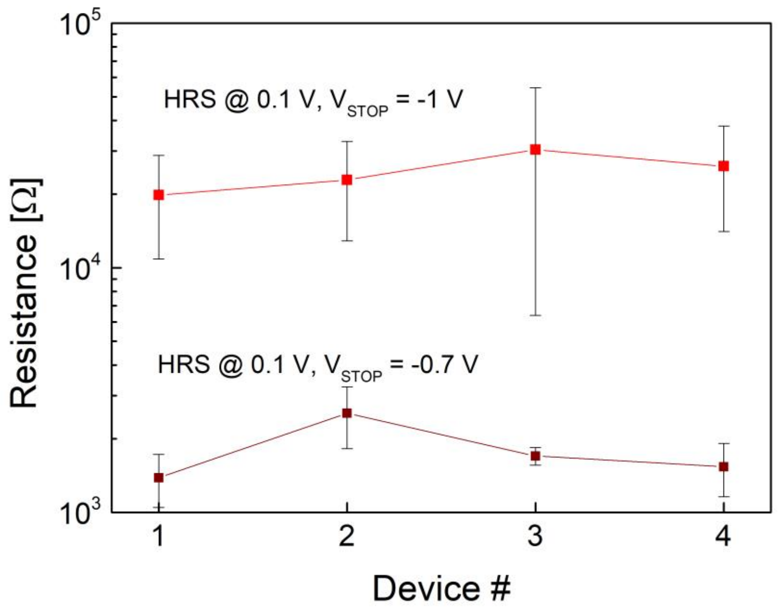

Figure. S1. The variation of the resistances of four Ag/a-SVO/Pt-based devices after 10 cycles.

2. Pulse measurement

The pulse measurement was carried out to evaluate the synaptic behavior of Ag/a-SVO/Pt memristor. Schematic diagram for monitoring the transient response by the oscilloscope in parallel with the shunt resistor (load resistor) is shown in Fig. S3. The conductance of Ag/a-SVO/Pt memristor was obtained by monitoring the voltage change in the shunt resistor.

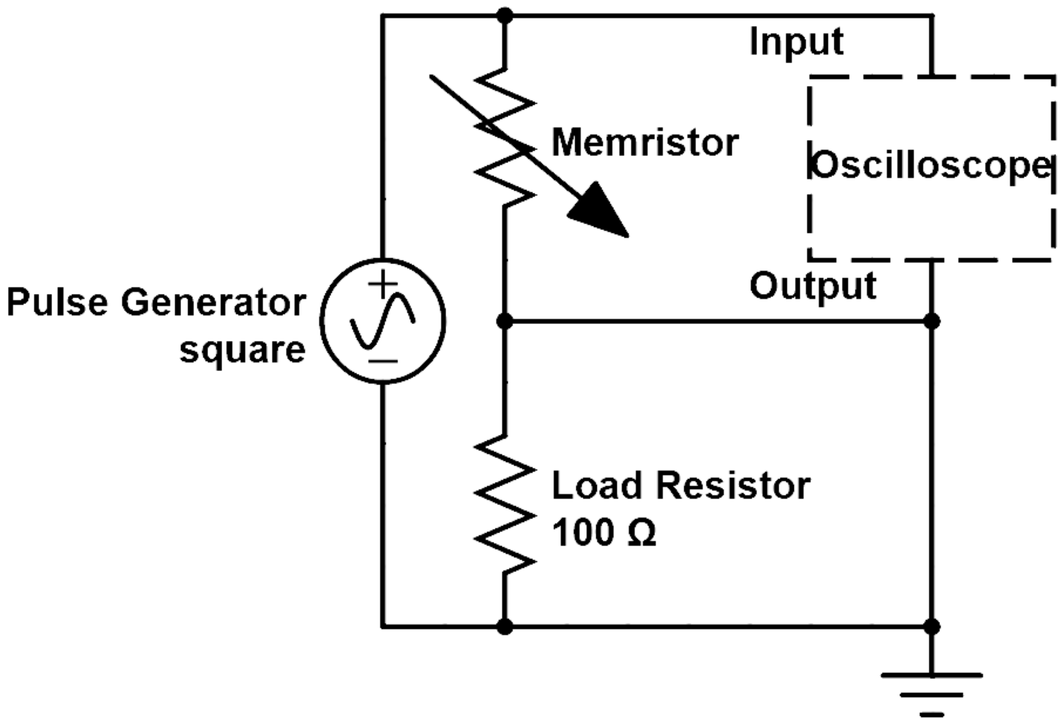

Figure. S2. Schematic diagram of a setup for the electrical characterization of the memristor.

3. Nonlinearity calculation

The number of potentiation/depression pulses changes the conductance of the memristor. This conductance changes are not identical. This non-ideal effect could be quantitatively calculated using a nonlinearity factor as shown below:

$$\mathrm{NL} = \left| \frac{\left[{(G_{max}-G_{min})}/{2} + G_{min} \right] - G_S}{{(G_{max}-G_{min})}/{2} + G_{min}} \right| \times 100\ \% \qquad (1)$$

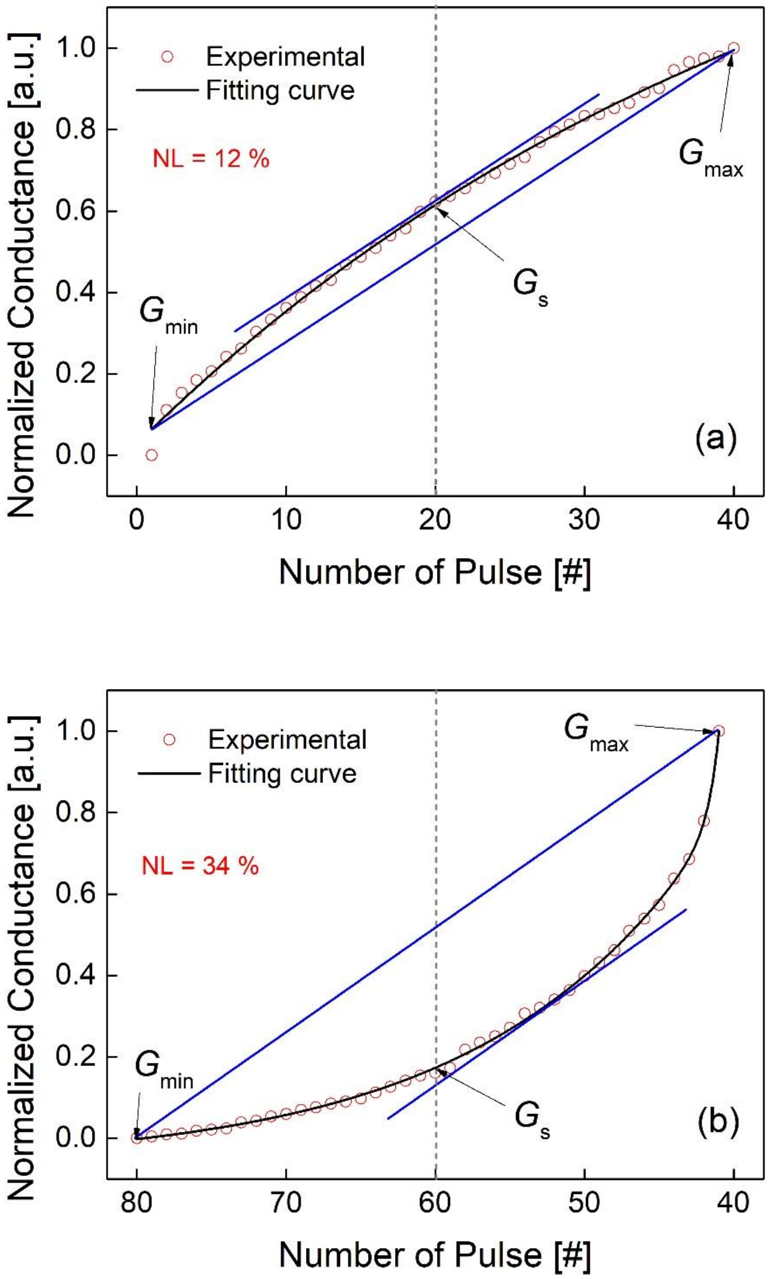

Figure. S3. Nonlinearity calculation of Ag/a-SVO/Pt memristors (a) in depression and (b) potentiation.

4. Reverse bias

To confirm the possibility of the effect of oxygen vacancy migration/recombination, a DC reverse bias sweep is applied at Ag/a-SVO/Pt memristor. DC voltage sweeping from 0 V → -4 V → 0 V with an *I*cc of 1 μA, which did not result in a SET process. If oxygen vacancies were generated and migrated toward the bottom Pt electrode, the conduction filaments should be formed and current values be not the same during DC sweeping from 0 V → -4 V → 0 V. Because the resistance state was not changed at potential differences within 4 V, it can be envisaged that oxygen vacancies migration did not occur. Consequently, the conduction filament of the Ag/a-SVO/Pt memristor was formed via Ag^+ migration under the external electric field.

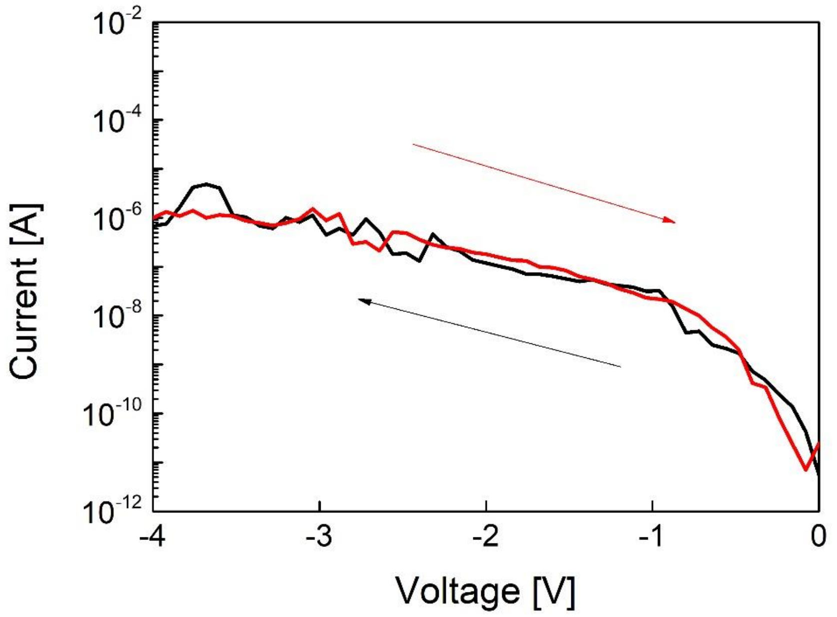

Figure. S4. The *I*-*V* curves of the Ag/a-SVO/Pt memristor at reverse biases.

Table S1. Comparison of our devices and other devices.

Type	Structure	Active ions	Pulse width	# of P/D pulses	G_{max}/G_{min}
Filamentary	Ag/SVO/Pt [This work]	Ag	100 ns	40/40	~ 4.3
Filamentary	$Ta/TaO_x/TiO_2/Ti$[S1]	V_O	5 ms	50/50	~ 1.5
Filamentary	$TiN/TEL/HfO_x/TiN$[S2]	V_O	50 ns	200/100	~ 10
Ferroelectric	Pt/BTO/SNTO[S3]	-	50 ns	100/100	~ 12
Phase change	$Ge_2Sb_2Te_5$[S4]	-	50 ns	20/20	~ 270
Filamentary	Ag/SiGe/p-Si[S5]	Ag	5 μs	500/500	~ 240
Ferroelectric	$TiN/Hf_{0.5}Zr_{0.5}O_2/Pt$[S6]	-	100 ns	38/38	~ 4

Reference

S1. Wang, I. T., Chang, C. C., Chiu, L. W., Chou, T. & Hou, T. H. 3D Ta/TaO_x /TiO_2/Ti synaptic array and linearity tuning of weight update for hardware neural network applications. *Nanotechnology* **27**, 365204, doi:10.1088/0957-4484/27/36/365204 (2016).

S2. Wu, W. et al. Improving Analog Switching in HfO_x-Based Resistive Memory With a Thermal Enhanced Layer. *IEEE Electron Device Lett.* **38**, 1019-1022, doi:10.1109/led.2017.2719161 (2017).

S3. Li, J. et al. Reproducible Ultrathin Ferroelectric Domain Switching for High-Performance Neuromorphic Computing. *Adv. Mater.* **32**, e1905764, doi:10.1002/adma.201905764 (2020).

S4. Nandakumar, S. R. et al. A phase-change memory model for neuromorphic computing. *J. Appl. Phys.* **124**, 152135, doi:10.1063/1.5042408 (2018).

S5. Choi, S. *et al.* SiGe epitaxial memory for neuromorphic computing with reproducible high performance based on engineered dislocations. *Nat. Mater.* **17**, 335-340, doi:10.1038/s41563-017-0001-5 (2018).

S6. Chen, L. *et al.* Ultra-low power $Hf_{0.5}Zr_{0.5}O_2$ based ferroelectric tunnel junction synapses for hardware neural network applications. *Nanoscale* **10**, 15826-15833, doi:10.1039/c8nr04734k (2018).
